# Comparison of Human Premixed and Basal Plus Short-Acting Insulin Regimens for Individuals With Type 2 Diabetes During Ramadan Fasting

**DOI:** 10.7759/cureus.11976

**Published:** 2020-12-08

**Authors:** Mahmood Thamer Altemimi, Samih A Odhaib, Husam J Imran, Ali Alhamza, Ammar Almomin, Abbas A Mansour

**Affiliations:** 1 Adult Endocrinology, Faiha Specialized Diabetes, Endocrine and Metabolism Center, University of Basrah, Basrah Health Directorate, Basrah, IRQ

**Keywords:** type 2 diabetes mellitus, ramadan, fasting, premixed insulin, basal-plus short-acting insulin, insulin regimen

## Abstract

Background

Premixed insulin and basal insulin plus short-acting insulin regimens may be of value for treating individuals with type 2 diabetes (T2DM) who are fasting during Ramadan due to simplicity and better compliance. The objective of this study was to compare the effectiveness of human premixed insulin to basal plus short-acting insulin regimens in the management of fasting individuals with T2DM during Ramadan.

Methods

We conducted a prospective observational study in Basrah (southern Iraq) on 30 individuals with T2DM who fast during Ramadan. The enrolled patients were assigned into two groups at random: one group received a human premixed insulin regimen, the other received a basal plus short-acting insulin regimen. A baseline clinical and biochemical analysis was gathered for all patients at recruitment two weeks before fasting and within four weeks after the end of fasting. Patients were assessed twice during fasting month for insulin dose adjustment and documentation for any hyperglycemia or hypoglycemia.

Results

Fourteen patients were assigned to the premixed group, and 16 patients were assigned to the basal plus short-acting insulin group. The mean patient age was 53 ± 8 years, and the mean T2DM duration was 9.3 ± 4.2 years. The two groups were matched by age, body mass index, and glycated hemoglobin (HbA1c). There was no significant difference between the initial and final mean HbA1c in both groups. However, there was more non-significant HbA1c reduction in the premixed group as compared to the basal plus short-acting insulin group. The number of hypoglycemic events and weight changes among the two groups was not significant.

Conclusions

Both human premixed and basal plus short-acting insulin regimens are equally useful for glycemic control for patients with T2DM who choose to fast in observance of the month-long holiday of Ramadan.

## Introduction

The management of Muslim individuals with type 2 diabetes mellitus (T2DM) who fast during Ramadan represents a challenge for health care professionals, given the potential risks of hypoglycemia, hyperglycemia, diabetic ketoacidosis, and dehydration [1]. Limited data are available regarding the optimal insulin type or regimen for people with T2DM during Ramadan, but results from several studies indicate that appropriate modification and individualization of insulin regimens are required [2-7]. The available evidence suggests that both basal plus short-acting insulin and premixed insulin are comparable in terms of safety and efficacy when used for insulin initiation in insulin-naive patients and intensiﬁcation in patients for whom basal insulin failed. These two simple regimens may be of value for treating patients with T2DM who intend to fast during Ramadan for their relatively good compliance [8]. This study was conducted to compare the degree of glycemic control, tolerability, and the existence of dysglycemic events whether (hypo and/or hyperglycemia) from the use of either human premixed insulin or basal plus short-acting insulin regimens during treating patients with T2DM who intend to fast Ramadan.

## Materials and methods

Thirty individuals with T2DM, mean age 53 ± 8 years, attended Faiha Specialized Diabetes Endocrine and Metabolism Center (FDEMC) in Basrah (southern Iraq) for glycemic control and assessment. Those individuals were willing to fast Ramadan without evident contraindication to their fasting, and fourteen of them were already treated by human premixed insulin (70/30; consisting of both neutral protamine Hagedorn [NPH] insulin in 70% and human regular short-acting insulin in 30%) regimen before Ramadan (labeled as group one) while the remaining sixteen patients were treated by human basal (NPH) insulin with or without a single bolus dose of human regular short-acting insulin prior major meal before Ramadan (labeled as group two). During Ramadan, all individuals were willing to fast without contraindication and sharing the standard of consuming only two meals each day: iftar (the sunset meal at 6:30 pm) and suhoor (the predawn meal at 3:00 am).

Group one was treated by a human premixed insulin (NPH/regular 70/30 regimen in a dose of 0.5-1 unit/kilogram daily; two-thirds of the dose before iftar and one-third before suhoor) and instructed to titrate their dose of insulin accordingly. Group two was treated by basal plus short-acting insulin regimen in a dose of 0.5-1 unit/kilogram daily (half the dose as human regular short-acting insulin before iftar, and the other half of the dose as basal NPH insulin during the midnight). These insulin doses were adjusted regularly according to calorie intake and regular self-monitoring of blood glucose (SMBG). All patients were given 1000 mg of metformin twice daily with their insulin regimens during the study.

At the recruitment visit two weeks before fasting, participants provided informed written consent. Patients’ medical history was taken and examined for weight, body mass index (BMI; kg/m^2^), and blood pressure (mmHg). They were provided proper instructions for insulin dose adjustment, diet control, regular SMBG at least three times daily, and documentation of any hypoglycemia (defined as blood glucose < 70 mg/dL) or hyperglycemia (defined as blood glucose > 300mg/dL) which is permitting to break fasting at that day [7]. 

At the second visit (which occurred during the first week of Ramadan) and the third visit (which occurred two weeks later), the patients were re-evaluated for insulin dose adjustments according to their SMBG level, and they were assessed for any episodes of hypoglycemia and hyperglycemia.

The fourth visit (i.e., the last visit) occurred four weeks after the end of Ramadan, to avoid any possible recall bias regarding missing information, in which information retrieval was done via a preformed questionnaire, to register hypoglycemic or hyperglycemic events, tolerability, SMBG reading, and any other self-reported events. Accordingly, changes in patients’ parameters between the two groups were evaluated regarding weight, BMI, glycated hemoglobin (HbA1c), hypoglycemia, hyperglycemia, and breaking the fast.

Samples of venous blood were taken during the first and last visit for HbA1c evaluation and fasting plasma glucose, and the samples were sent immediately to the laboratory for assessment (COBAS INTEGRA® 400 Plus, Roche Diagnostics, Basel, Switzerland).

Data were analyzed using IBM SPSS Statistics for Windows, Version 23.0 (IBM Inc., Armonk, USA). The parametric variables were presented in the form of mean ± standard deviation (SD), mean ± standard error (SE), or frequency (%) for data expression. A dependent t-test for continuous parameters of both groups was used, and a p-value <0.05 was considered to be statistically significant at a 95% confidence interval (CI).

## Results

Table 1 represents the different demographic characteristics of patients in both groups. The mean age for the enrolled patients was 53 ± 8 years (51 ± 10 years for the premixed group and 54 ± 6 years for the basal plus short-acting group). The male-to-female ratio was approximately 1:1. The mean duration of T2DM was 9.3 ± 4.2 years (8.8 ± 4 years in the premixed group and 9.8 ± 4.5 years in the basal plus short-acting group).

**Table 1:** Demographic characteristics of the enrolled patients with type 2 diabetes on either premixed or basal plus rapid-acting insulin regimens BMI - body mass index; HbA1c - glycated hemoglobin; SD - standard deviation; T2D - type 2 diabetes

Variables		Premixed regimen	Basal plus short-acting regimen	Total	p
Age (years)	mean ± SD	51.2 ± 10.48	54.6 ± 6.35	53 ± 8.5	0.284
Gender	Women, n (%)	9 (64.3%)	7 (43.8%)	16	0.276
	Men, n (%)	5 (35.7%)	9 (56.3%)	14	
Duration of T2D (years)		8.8 ± 4	9.8 ± 4.5	9.3 ± 4.27	0.55
Initial weight (kg)		79.15 ± 12.18	87.2 ± 13.6	83.6 ± 13.4	0.106
Last weight (kg)		79.07 ± 11.63	86.87 ± 14.8	83.37 ± 13.82	0.133
Initial BMI (kg/m^2^)		31.3 ± 5.16	31.7 ± 5.04	31.5 ± 5	0.845
Last BMI (kg/m^2^)		31.34 ± 4.11	31.61 ± 5.41	31.49 ± 4.79	0.883
Initial HbA1c (%)		10.4 ± 2	10.4 ± 2.6	10.4 ± 2.3	0.993
Last HbA1c (%)		8.4 ± 1.49	8.83 ± 1.54	8.6 ± 1.51	0.447
Creatinine (mg/dL)		0.78 ± 0.25	1.1 ± 1.3	0.9 ± 1.01	0.401
Total		14 (46.3%)	16 (53.7%)	30	

**Figure 1:**
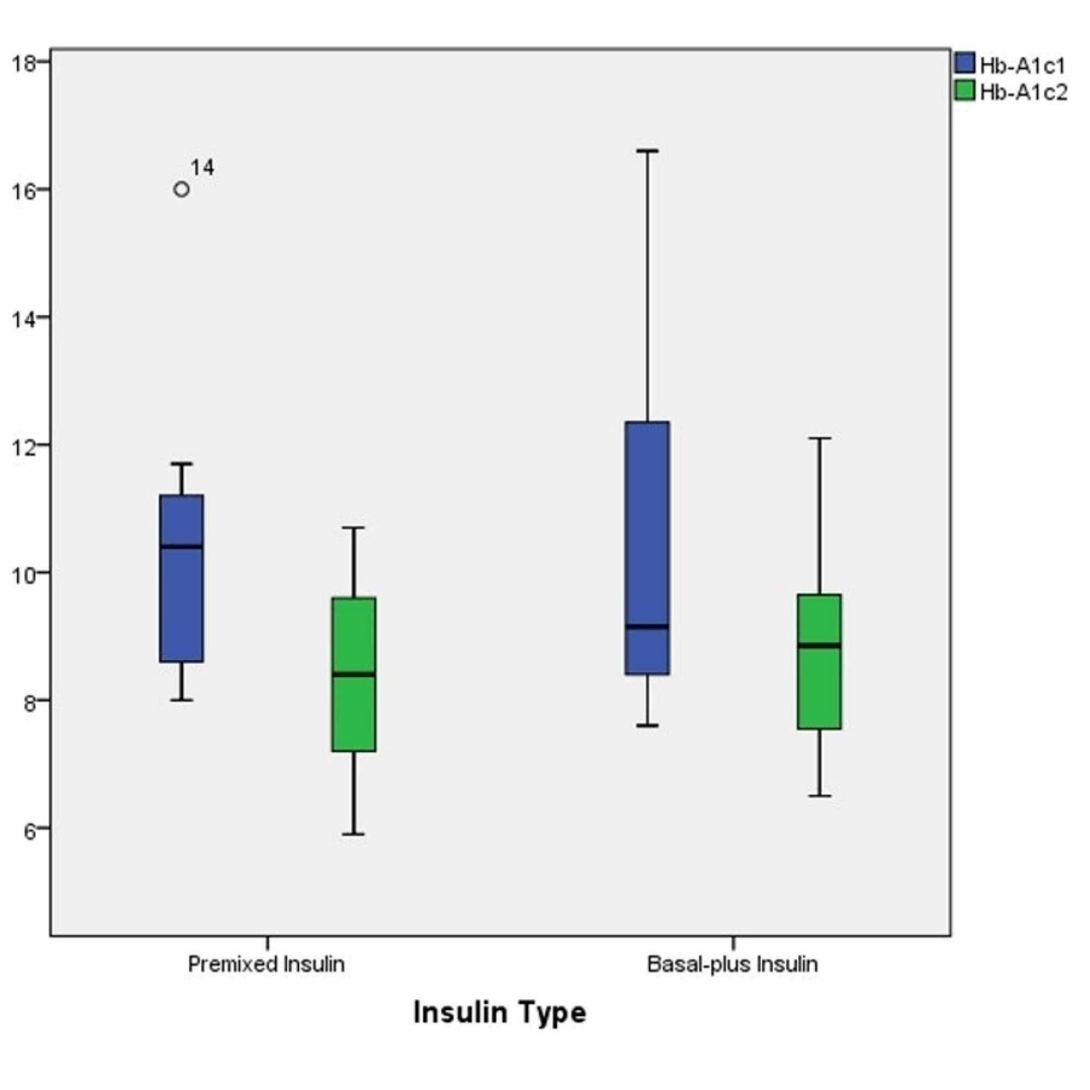
Box and Whisker-plot illustrate the interquartile range of both initial and last glycated hemoglobin among patients with type 2 diabetes treated with either human premixed or basal plus short-acting insulin regimens

The degree of glycemic control was assessed by the change in HbA1c between last and initial visits (delta HbA1c) among all patients. There was a significant difference between the initial and the last HbA1c within the same group of either premixed regimen or basal plus short-acting insulin regimen, but this difference was non-significant between the two groups (Table 2, Figure 1).

**Table 2:** The effect of insulin type on the changes of both glycated hemoglobin and weight CI - confidence interval; HbA1c - glycated hemoglobin; SE - standard error

Variables		Premixed regimen	Basal plus short-acting regimen	p
Delta HbA1c (%)	mean ± SE	2 ± 0.387	1.56 ± 0.431	0.462
	P	< 0.0001	0.003	
	95% CI	(1.16 - 2.83)	(0.642 - 2.48)	(-1.64 - 0.76)
Delta weight (kg)	mean ± SE	0.07 ± 1.28	0.4 ± 0.81	0.824
	P	0.953	0.627	
	95% CI	(-2.7 - 2.8)	(-1.33 - 2.15)	(-2.68 - 3.34)

Another parameter for the glycemic control was the occurrence of hypoglycemia and hyperglycemia, with some degree of intolerability to both insulin regimens, and breaking the fast as an ultimate measure. Table 3 demonstrated that even with the existence of dysglycemic events in patients of both groups, the difference in the prevalence of hypoglycemia or hyperglycemia was non-significant. Tables 1-2 demonstrated that the differences in weight and BMI between the first and last visits were non-significant within the same group and between the two groups.

**Table 3:** Comparative clinical characteristics of patients with type 2 diabetes on either premixed or basal plus rapid-acting insulin regimens

Variables		Premixed regimen	Basal plus short-acting regimen	P
Hypoglycemic events, n (%)	No	9 (64.3%)	9 (56.3%)	0.654
	Yes	5 (35.7%)	7 (43.8%)	
Hyperglycemic events, n (%)	No	5 (35.7%)	6 (37.5%)	0.919
	Yes	9 (64.3%)	10 (62.5%)	
Breaking the fast, n (%)	No	12 (85.7%)	13 (81.25%)	0.571
	Yes	2 (14.28%)	3 (18.75%)	
Total		14	16	

## Discussion

In many Muslim countries, premixed insulin is one of the most frequently prescribed formulations in patients with T2DM. However, most patients will require rapid- or short-acting insulin administered in combination with the basal insulin at meals, particularly at the evening meal, which typically contains a larger caloric load [1].

Despite the small number of patients with T2DM successfully recruited for this study to complete the fasting for about 30 days of Ramadan, the demographic and clinical characteristics of patients in both insulin regimens were nearly matched regarding age and diabetes duration, HbA1c, body weights, and BMI. Both types of insulin regimens have an efficient glycemic reduction as assessed by HbA1c level, but it was more evident with human premixed insulin, which was similar to that reported by Downie et al. [8].

The achievement of such glycemic control by the two insulin regimens could be explained by the frequent follow up during the four visits, which included the adjustment of the dietary and insulin dosing, which was also ascertained by other studies [9-13].

The time elapsed during the study was not enough to achieve any significant weight reduction from the baseline weight and BMI. Bearing in mind that insulin may cause some weight gain rather than loss, dietary changes during the relatively short period of the study would have a low level of impact. Many other studies demonstrated the same assumption [9-13].

Some of the challenges faced by T2DM patients who fast during Ramadan are hypoglycemic and hyperglycemic events that render the breaking of the fast a near possibility. We registered more hypoglycemic events in the basal plus short-acting insulin regimen group more than the premixed group, but with no significant difference. The high variability of the basal component of the basal plus short-acting regimen could be implicated in the causation and may favor the glycemic control achieved by the premixed insulin regimen as a better option. However, these are often accompanied by weight gain and/or hypoglycemia, and results should be interpreted within the context of total insulin doses used [8]. In addition to fasting duration between iftar and suhoor is varied widely across the world. The type of this short-timed study and the small number of enrolled patients limit the generalizability of the results.

## Conclusions

Both human premixed and basal plus short-acting insulin regimens are effective for glycemic control for people with T2DM who intend to fast during Ramadan. The Muslim individuals on these two regimens can fast safely if the treatment is personalized on a case-by-case basis.
